# Interferon-Stimulated Gene Expression in Peripheral Blood Leucocytes as a Convenient Prediction Marker for Embryo Status in Embryo-Transferred Japanese Black Cows during the Peri-Implantation Period

**DOI:** 10.3390/vetsci10070408

**Published:** 2023-06-22

**Authors:** Hitomi Yoshino, Keiichiro Kizaki, Toh-ichi Hirata, Kosuke Iga, Hideo Matsuda, Tadayuki Yamanouchi, Yutaka Hashiyada, Kei Imai, Toshina Ishiguro-Oonuma, Tomomi Kanazawa, Toru Takahashi, Kazuyoshi Hashizume

**Affiliations:** 1 Cooperative Department of Veterinary Medicine, Faculty of Agriculture, Iwate University, 3-18-8 Ueda, Morioka 020-8550, Iwate, Japan; yoshino1990710@gmail.com (H.Y.); oonumat@iwate-u.ac.jp (T.I.-O.); tomomik@iwate-u.ac.jp (T.K.); tatoru@iwate-u.ac.jp (T.T.); kazuha@iwate-u.ac.jp (K.H.); 2 Field Science Center, Faculty of Agriculture, Iwate University, 3-18-8 Ueda, Morioka 020-8550, Iwate, Japan; hirata@iwate-u.ac.jp; 3 Tohoku Agricultural Research Center, National Agriculture and Food Research Organization, 4 Akahira, Shimo-kuriyagawa, Morioka 020-0198, Iwate, Japan; kosukei@affrc.go.jp; 4 National Livestock Breeding Center, Nishigo 961-8061, Fukushima, Japan; h0matsud@nlbc.go.jp (H.M.); t0yamano@nlbc.go.jp (T.Y.); yhashiy@ishikawa-pu.ac.jp (Y.H.); 5 Department of Sustainable Agriculture, Rakuno Gakuen University, 582 Bunkyodai-Midorimachi, Ebetsu 069-8501, Hokkaido, Japan; imai@rakuno.ac.jp

**Keywords:** pregnancy diagnosis, gestational status, biomarker

## Abstract

**Simple Summary:**

Practically and economically, predicting pregnancy status is crucial for food animal production. Recently, the expression of interferon-stimulated genes (ISGs) in peripheral blood leukocytes has been examined as a biomarker for predicting the gestational status during maternal recognition in ruminants. Although the ISG levels in the blood cells of cattle have been reported, the practical application of this detection method in embryo-transferred (ET) cows has not been evaluated in detail. The levels of three of the four ISGs in this study were significantly higher in pregnant cows than in non-pregnant cows on day 21 of gestation. The diagnostic performance results showed that the three ISGs are appropriate biomarkers for predicting the gestational status of ET cows. The statistical evaluation of diagnostic accuracy in ET cows indicated that the values of sensitivity, specificity, and accuracy were very high and the positive predictive values of the three ISGs were estimated. The results of this study suggest that ISGs are good biomarkers for gestational status during the peri-implantation period in ET cattle.

**Abstract:**

Pregnancy diagnosis during early gestation is important for cattle reproduction. The expression of interferon-stimulated genes (ISGs) in peripheral blood leukocytes (PBLs) was studied in embryo-transferred (ET) Japanese Black cattle. ISGs in PBLs—*ISG15*, *MX1*, *MX2*, and *OAS1*—were detected in multiple ovulation ET cattle using a real-time quantitative polymerase chain reaction, and receiver operating characteristic (ROC) curve analysis was performed. Gestational status was predicted using the average ISG levels during the normal estrous cycle (AVE) and the Youden index from the ROC curve analysis as cutoff values. The *ISG15*, *MX1*, and *MX2* levels were significantly higher in pregnant cattle (*n* = 10) than in non-pregnant cattle (*n* = 23) on gestation day 21, whereas the levels of all ISGs were similar between non-pregnant and non-pregnant cattle with late embryonic death (*n* = 7). *ISG15*, *MX1*, and *MX2* appropriately predicted the gestational status of ET cows. The statistical evaluation of the diagnostic accuracy in ET cows on day 21 of gestation presented higher values of sensitivity, specificity, accuracy, and positive predictive values of *ISG15, MX1*, and *MX2* using the Youden index than using the AVE. Therefore, *ISG15*, *MX1*, and *MX2* are excellent biomarkers of gestational status during the peri-implantation period in ET cattle.

## 1. Introduction

Interferon-tau (IFNT) is a unique molecule derived from trophoblastic cells in ruminants and is specifically produced during the early gestational period [1,2,3]. IFNT exerts an anti-luteolytic effect on the corpus luteum by attenuating the secretion of prostaglandin F2 alpha (PGF2 alpha) in the endometrium, and PGF2 alpha secretion is mediated by the downregulation of oxytocin receptors in uterine epithelial cells [1,2,3]. IFNT not only stimulates endometrial and luteal cells but also other cells, such as hepatocytes and blood cells [3,4,5,6,7]. It recognizes gestation and plays an immunological role in preventing infections and embryo–maternal interactions [8,9]. It plays a crucial role in the regulation of embryonic and endometrial factors during early gestation in ruminants. 

Interferon-stimulated genes (ISGs) are induced in response to interferon (IFN) [10]. IFNT, a type I IFN, binds to the cell-surface heterodimeric interferon alpha and beta receptor (IFNAR) 1/IFNAR2 complex [3]. IFNT signaling, through the Janus kinase/signal transducer and the activator of the transcription (JAK/STAT) pathway, involves a transcriptional heterotrimer consisting of phosphorylated STAT1/STAT2 and interferon regulatory factor (IRF9) [3]. Activated interferon regulatory factor 3, which consists of phosphorylated STAT1/STAT2 and IRF9, translocates to the nucleus and promotes gene transcription by binding to IFN-stimulated response elements in the upstream promoter regions of ISGs [11]. It was previously considered that there are several hundred ISGs; however, in recent years, it has been predicted that approximately 10% of the genes in the genome are regulated by IFN [12]. ISG15 ubiquitin-like modifier (*ISG15*), MX dynamin-like GTPase 1 (*MX1*), *MX2*, and 2’–5’ oligoadenylate synthetase 1 (*OAS1*), which are the focus of the present study, are typical ISGs [11]. ISGs and their related molecules coordinate the establishment of feto–maternal interactions and simultaneously stimulate blood cells. This specific response could be beneficial for predicting the gestational status of cattle during the early peri-implantation period, because IFNT plays specific roles in ruminants [2]. These complex events appear to be closely related to ISG expression in blood cells during maternal recognition.

Predicting pregnancy status is essential for animal food production, both practically and economically. Various methods of gestational prediction, including rectal palpation, ultrasonography, blood progesterone level measurement, and estrus detection, have been used to address this critical issue in cattle [13,14,15]. The detection of return to estrus on days 18–32 post-artificial insemination (AI) is the easiest method for identifying non-pregnant cattle [13]; however, this method is not sufficiently accurate for pregnancy diagnosis owing to its low efficiency (<50%) [16]. Diagnosis based on the rectal palpation of the ovary and uterus is the most widely used method, and it is performed correctly as early as 35 days post-AI [13,14]. Recently, portable brightness (B)-mode ultrasonography has been reported to be a widespread method, and it has been used to correctly detect early pregnancy 27 days post-AI under real-world conditions [13,17]. Moreover, indirect methods, such as the measurement of progesterone concentrations and pregnancy-associated glycoprotein (PAG) levels in milk and blood, have been developed [13,14,15]. Profiling progesterone concentrations on days 18–24 post-AI could be an effective method for identifying non-pregnant cattle [13]. However, non-pregnant cattle with an extended luteal phase are diagnosed as pregnant. Thus, progesterone tests are inadequate for the identification of pregnant cattle [13]. PAGs are secreted by the binucleated trophoblast cells in the placenta, and their levels increase from 15 to 35 days of gestation in cattle [13,18]. Assay kits for milk and blood PAG levels have recently been commercialized. The PAG levels in milk and blood are reliable indicators for pregnancy diagnosis approximately 26–30 days post-AI [13,14]. 

In the above-mentioned studies, correct diagnosis was made after the period of normal return to estrus, and thus, early rebreeding opportunities were probably lost in non-pregnant cattle. Therefore, establishing pregnancy diagnosis methods before the end of the estrous cycle is required to improve reproductive efficiency. In addition, the detection and prediction of pregnancy loss (late embryonic death or abortion) after a single early pregnancy diagnosis are difficult; thus, multiple examinations are required to confirm pregnancy status. However, IFNT is a specific molecule that is difficult to use as a gestation prediction signal during the early implantation periods; that being said, it plays a role later on [1,2,19]. Recently, ISG expression in peripheral blood leukocytes (PBLs) has been examined as a biomarker for predicting the gestational status during maternal recognition in ruminants [20,21,22]. The prediction efficiency of ISGs, specifically *ISG15* and *MX2*, for the establishment of gestation has been confirmed as reliable biomarkers in cattle [21,23,24,25,26]. 

Embryo transfer is an assisted reproductive technology that is distinct from AI and has been developed and used in the food and livestock industries [14,27]. Embryo transfer is a method used to promote livestock improvement and produce animals with a high market value; however, the conception rate remains to be improved because only around half of embryo transfers succeed in practice [28,29,30]. In embryo transfer, as in AI, early pregnancy diagnosis, especially the identification of non-pregnant cattle, increases the chance of insemination and shortens the calving interval, consequently improving reproductive efficiency. *ISG15* expression in PBLs, a biomarker for predicting gestational status during maternal recognition, has been reported to increase in embryo transferred (ET) cows [31,32]. However, the practical applications of this detection method have not yet been evaluated in detail.

Therefore, in this study, we aimed to clarify the expression patterns of four ISGs [*ISG15*, *MX1*, *MX2*, and *OAS1*] in the PBLs of multiple ovulation embryo transfer (MOET) cattle on day 21 after estrus (14 days after embryo transfer) and to verify a method for predicting pregnancy status in ET Japanese Black (JB) cattle during the peri-implantation period.

## 2. Materials and Methods

### 2.1. Animals

The Iwate University Laboratory Animal Care and Use Committee approved the experimental and feeding conditions of the cattle used in this study (approval numbers: A201244, A201434, and A201701). Twenty-two heifers and eighteen parous JB cattle (*n* = 40) from an experimental farm were used in this study. Bovine blastocysts were produced on day 7 using the MOET technique [33]. Transferable embryos, identified according to the International Embryo Transfer Society manual as either code 1 or 2 single blastocysts on day 7 after insemination, were transferred to the uterine horn ipsilateral to the corpus luteum horn of each synchronized recipient on day 7 after estrus, as previously reported [34]. Recipient estrous behavior was observed at least twice daily, in the morning and evening. Peripheral blood was collected on day 21 of gestation (14 days after embryo transfer). The pregnancy status was confirmed on days 30 and 60 of gestation using ultrasonographic detection (UD). In the present study, three different categories of cows, namely, 10 pregnant ET cows, 23 non-pregnant cows, and 7 cows that were non-pregnant with late embryonic death, were included from the experimental farm. In pregnant ET cows, the conceptus was observed using UD at approximately 30 days (23 days after embryo transfer) and 60 days of gestation. Conversely, in non-pregnant ET cows, no conceptuses were observed at approximately 30 and 60 days. Pregnancy loss in cows in the present study occurred after a conceptus was observed on day 30 of gestation. Thus, the conceptus was observed on day 30 but not on day 60 in the late embryonic death group.

### 2.2. Sample Collection, RNA Extraction, and Real-Time Quantitative Polymerase Chain Reaction (RT-qPCR)

Blood collection, RNA extraction, and RT-qPCR were performed as previously described [26]. Approximately 3 mL of peripheral blood was collected in PAXgene blood RNA tubes (Qiagen, Hilden, Germany) and incubated at room temperature for 2 h. RNA was extracted using an RNA extraction kit (PAXgene Blood RNA Kit; Qiagen). RNA samples were evaluated using a NanoDrop spectrophotometer (ND-1000; Thermo Fisher Scientific, Waltham, MA, USA) and treated with DNase (TURBO DNA-free Kit; Ambion, Austin, TX, USA) to remove contaminating genomic DNA. Reverse transcription was performed using 1 µg of total RNA with random primers and a high-capacity reverse transcription kit (Applied Biosystems, Foster City, CA, USA), according to the manufacturer’s instructions. The reverse transcription cycle was performed at 25 °C for 10 min, 37 °C for 120 min, and 85 °C for 5 s in a thermal cycler. PCR analysis was performed using the Power SYBR Green PCR Master Mix (Thermo Fisher Scientific) on the ABI7300 real-time PCR system (Applied Biosystems), as previously described [24]. The primers used to amplify each gene are listed in Table 1 [24]. The amplification conditions were as follows: initial sample incubation at 50 °C for 2 min and 95 °C for 10 min, followed by 40 cycles at 95 °C for 15 s and 60 °C for 1 min, with the collection of fluorescence signals at the end of each cycle. The melting curve for detecting the SYBR Green-based objective amplicon was confirmed at 65 to 95 °C in 0.5 °C increments. To quantify the concentration of each mRNA, standard curves were generated via the serial dilution of a plasmid containing the corresponding cDNA, as previously described [22]. For standard plasmid preparation, PCR fragments were subcloned into the pGEM-T Easy Vector System (Promega, Madison, WI, USA), according to the manufacturer’s instructions, and the sequences of the cloned plasmid containing cDNA were analyzed using the ABI PRISM 3100-Avant Genetic Analyzer (Applied Biosystems). In addition, the mRNA expression of glyceraldehyde-3-phosphate dehydrogenase (*GAPDH*) was used as a reference gene for the normalization of internal expression in the samples. The expression of beta-actin and ribosomal protein L27, in addition to that of *GAPDH*, was also examined to evaluate their use as reference genes for RT-qPCR. The results showed no significant differences in their expression. Quantitative PCR was performed according to the Minimum Information for Publication of Quantitative Real-Time PCR Experiments (MIQE) guidelines [35]. 

### 2.3. Estimation of Threshold Values

The average values (AVE) of normalized ISG expression levels during the estrous cycle in JB cattle have been previously reported [26] as follows: *ISG15*, 0.174; *MX1*, 0.065; *MX2*, 0.098; and *OAS1*, 0.351. ISG expression levels on day 21 of gestation in ET cows were used to construct a receiver operating characteristic (ROC) curve. According to the RT-qPCR experiments in the present study, the reference cDNA for *ISG15*, *MX1*, *MX2*, and *OAS1* and cDNA sample synthesized from PBL RNA were measured simultaneously to allow comparison with AVE values obtained in previous studies. The Youden index and area under the ROC curve (AUC) were estimated using JMP software (SAS Institute Inc., Cary, NC, USA). The AVE and Youden index were used as threshold values to predict the accuracy of pregnancy diagnosis in ET cows on day 21 of gestation. The results were as follows: true positive (TP), where the pregnancy status was determined as positive using the cutoff value of AVE or the Youden index and with the use of UD at around 60 days of gestation; false positive (FP), where the pregnancy status was determined as positive using the cutoff value of AVE or the Youden index but negative using UD; true negative (TN), where the pregnancy status was determined as negative using both cutoff value of AVE or the Youden index and UD; and false negative (FN), where the pregnancy status was determined as negative using the cutoff value of AVE or the Youden index but positive using UD. The sensitivity (TP/(TP + FN) × 100 (probability of a diagnosis of pregnancy among cows that were actually pregnant)), specificity )TN/(TN + FP) × 100 (probability that cows were diagnosed as non-pregnant)), positive predictive value (PPV; TP/(TP + FP) × 100), and negative predictive value (NPV; TN/(TN + FN) × 100) were calculated. The accuracy of pregnancy determination, (TP + TN)/(TP + FP + TN + FN) × 100, was evaluated by calculating the diagnostic accuracy. 

### 2.4. Statistical Analysis

Gene expression was measured using RT-qPCR and analyzed using Kruskal–Wallis test, followed by Steel–Dwass test, using JMP 7 software. Results with *p* < 0.05 were considered significant.

## 3. Results

### 3.1. Classical ISG Expression in ET Cattle and ROC Curve Analysis

The expression of *ISG15*, *MX1*, and *MX2* was significantly higher in pregnant cattle than in non-pregnant cattle on day 21 of gestation; however, no differences were observed in *OAS1* expression (Table 2). The expression of most ISGs was similar between the non-pregnant and late embryonic death groups.

**Figure 1:**
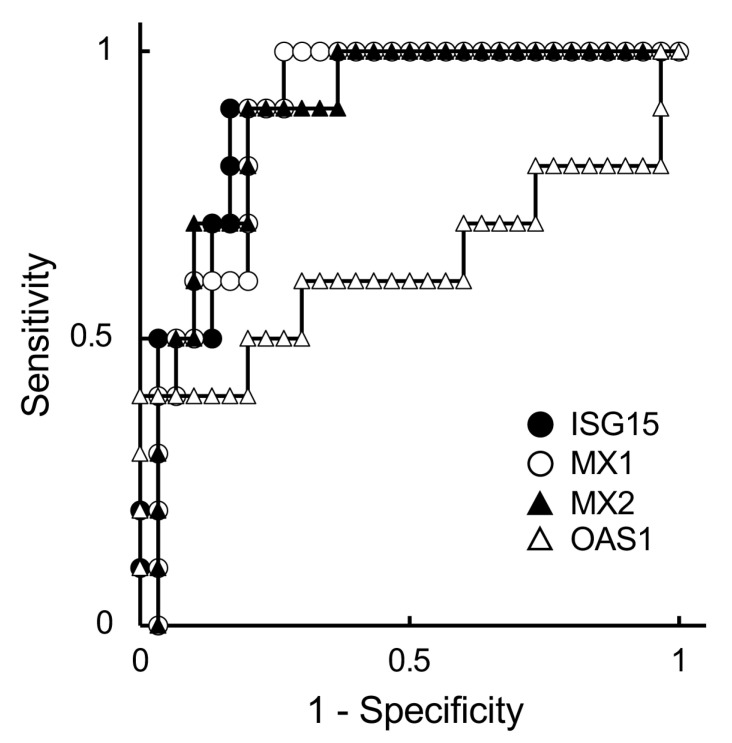
Receiver operating characteristic (ROC) curves of expression of ISGs in embryo-transferred JB cattle on day 21 of gestation. The expressions of *ISG15* (closed circle), *MX1* (open circle), *MX2* (closed triangle), and *OAS1* (open triangle) were analyzed using ROC curves. The horizontal and vertical axes are 1-specificity and sensitivity, respectively. Therefore, the upper-left corner is the ideal point with 100% sensitivity and specificity.

To determine a more suitable predictive marker for embryonic status, specifically in ET cattle, ISG expression levels in PBL were estimated and evaluated using the ROC curve analysis (Figure 1 and Table 3). 

We used the values of the non-pregnant and non-pregnant with late embryonic death groups for ROC curve analysis because the ISG levels were not different between these groups (Table 2). The ROC curve showed that all ISG levels, except the *OAS1* level, appropriately predicted the gestational status on day 21 (Figure 1). The AUC values of ISGs were categorized as high or nearly high (Table 3). 

### 3.2. Prediction of Gestational Statuses during the Peri-Implantation Period in ET Cattle

The gestational status during the peri-implantation period in ET cattle was predicted using conventional cutoff values (AVE) for ISGs, which were estimated from a previous study [26], and the Youden index cutoff values were estimated using the ROC curve analysis (Table 3). When applying the AVE values of the ISGs to predict gestational status on day 21 of gestation, the sensitivity was relatively higher (60.0–100%), but the specificity and accuracy values (10.0–66.6%) were lower than those determined using the Youden index (Table 4). For *ISG15*, *MX1*, and *MX2*, the sensitivity, specificity, and accuracy of the Youden index were 80% or higher. Each NPV showed high values estimated using AVE and the Youden index methods. 

## 4. Discussion

Indicators of gestational prediction are practically and economically crucial for domestic cattle reproduction. We established the accuracy of ISG expression in PBLs as predictive evidence in artificially inseminated Holstein and JB cattle [25,26] and applied this method to JB cows in the current study. First, the expression of ISGs in PBLs was examined to determine the suitability of the practical application of blood ISG levels as indicators of gestation prediction during the peri-implantation phase. Ten pregnant, twenty-three non-pregnant, and seven non-pregnant cows with late embryonic death were used to predict the gestational status of ET cows using ISG values. The fertility rates of the flock were low but presented a convenient model for late embryonic loss and/or death. 

On day 21 of pregnancy, the levels of *ISG15*, *MX1*, and *MX2* in PBLs were considerably higher in the pregnant group than in the non-pregnant group, similar to those reported by previous studies on AI [25]. Therefore, the reliability of ISG cutoff values was assessed in ET cows to predict the gestational status during the peri-implantation interval. The Youden index has been used to estimate the cutoff values for different physiological and pathological statuses using ROC curves [29,30]. The use of previously reported AVE ISG cutoff values [19] and the reliability of this technique were confirmed using ROC and the Youden index analyses in the present study, and the application of AVE cutoff values confirmed the credibility of the method. Using the AVE in ET JB cattle on day 21 of gestation, high sensitivity and NPV were observed, but the specificity, accuracy, and PPV were low. The Youden index cutoff method had higher specificity and accuracy than the AVE estimation. The AUC, obtained using the ROC curve analysis, indicated the accuracy of these predictions with high AUC values [36,37]. The values of *ISG15*, *MX1*, and *MX2* on day 21 were approximately 0.9 in ET cows and were almost the same as those in previous studies on AI cattle [26]. In previous studies, the differences in ISG level, specifically *ISG15* and *MX2*, between pregnant and non-pregnant cattle implied that they could be prominent indicators for predicting the gestation status in artificially inseminated Holstein and JB cattle [25,26]. However, *MX1* is not a suitable indicator when applying the AVE cutoff value for prediction because of its low specificity, precision, and PPV. However, by applying the Youden index to the cutoff value, *MX1* can also be used as an appropriate indicator, equivalent to *ISG15* and *MX2*. Therefore, *ISG15*, *MX1*, and *MX2* may be suitable indicators of pregnancy in ET cattle.

Interestingly, similar blood ISG levels were observed in the non-pregnant and late embryonic death groups in the present study. These results pose a simple question: why was the gene expression different between pregnant and non-pregnant cattle with late embryonic death on day 21 of gestation? This question arises because both groups would have had a conceptus around day 21. The ISG levels may reflect embryonic morphological changes during the implantation process [38,39]. The ISG levels in the non-pregnant and late embryonic death groups were similar in ET cattle on day 21, which may indicate the embryo development status. Embryos at approximately 17–19 days of gestation significantly change their shapes and activities, and the expanded fetal membrane covers the entire endometrial surface during the critical period of implantation [3,38,40]. The discrepancy in ISG level between the pregnant and non-pregnant late embryonic death groups suggests insufficient IFNT secretion by the embryos in the late embryonic death group. 

In domestic cattle reproduction, the true fertility rate is crucial for farmers who expect their cows to become pregnant as soon as possible after calving. Although various new technologies and strategies, such as superovulation, in vitro fertilization, and embryo transfer, have been applied to improve lifetime production in the cattle industry over the last several decades, embryo loss or death after fertilization remains a major issue, especially at 3–4 weeks [19]. Novel and cutting-edge techniques, such as embryo transfer, would be helpful in improving fertility in not only cattle but also other mammalian species, including humans, and their successful management should be considered. The results of the current study showed that the conceptus was observed using ultrasonography at around 30 days in both pregnant and late embryonic death groups. However, the ISG levels of the late embryonic death group on day 21 were lower than those of the pregnant group. It is important to determine whether embryos are dead, alive, or developing well. This means that some indicators are needed to determine the embryonic conditions. We expect that ISG detection methods in PBL will be useful in solving challenging issues. Unlike positive prediction, the negative prediction of gestational status in embryo transfer was reliable and ISGs were detectable in PBLs 14 days after embryo transfer. The application of ISG expression in PBLs during the peri-implantation period will provide accurate information under these circumstances. However, the sample size in the current study was small and none of the data on different days of the peri-implantation period, except for day 21 in ET cattle, were sufficient for gestational prediction. Further studies using more animals around peri-implantation are necessary to confirm the reliability of this theory regarding high gene expression in ET cattle. 

There are several practical methods for evaluating gestational status during early gestation, including serum progesterone and pregnancy-associated protein B measurements, and transrectal palpation. However, the prediction of gestational status in ET cattle is still difficult during the implantation period [14,41,42]. Specific gene expression in PBLs may be a reliable predictive method for determining the pregnancy status. Although this procedure may be reliable because the ISG prediction method has been established and used in cattle and other ruminants, we do not have sufficient information to identify PBLs that respond to *ISG15*, *MX1*, *MX2*, and/or *OAS1* [22]. The association between the patterns of gene expression in PBLs and indicators of different physiological circumstances is clear [24,25,43,44]. Further studies on the specificity of each gene are required to understand the precise responses to particular ISG molecules and the detailed roles of ISGs in the development of early stage embryos.

## 5. Conclusions

Classical ISGs, specifically *ISG15*, *MX1*, and *MX2*, are excellent biomarkers for predicting the non-pregnancy status during the peri-implantation period in ET JB cattle. These biomarkers may be reliable for the negative prediction of gestation during embryo transfer but are less reliable for establishing positive predictions. In addition, the expression of ISGs in the PBLs provides early gestational information during the recognition of gestation, regardless of the conception method in cattle.

## Figures and Tables

**Table 1:** Primer sequences for RT-qPCR.

Gene	Accession Number	Strand	Sequence (5′–3′)
*ISG15*	NM_174366	Forward	GCAGACCAGTTCTGGCTGTCT
		Reverse	CCAGCGGGTGCTCATCAT
*MX1*	NM_173940	Forward	GAGGTGGACCCCCAAGGA
		Reverse	CCACCAGATCGGGCTTTGT
*MX2*	NM_173941	Forward	GGGCAGCGGAATCATCAC
		Reverse	CTCCCGCTTTGTCAGTTTCAG
*OAS1*	NM_178108	Forward	CCAAGTCAAACAAGCCATCGA
		Reverse	CACATCGGAAACACCTCTCCTT
*GAPDH*	NM_001034034	Forward	AAGGCCATCACCATCTTCCA
		Reverse	CCACCACATACTCAGCACCAGCAT

**Table 2:** Expression of interferon-stimulated genes (ISGs) in peripheral blood leukocytes (PBLs) of pregnant and non-pregnant embryo-transferred Japanese Black (JB) cattle on day 21 of gestation (14 days after embryo transfer).

Group	*n*	*ISG15*	*MX1*	*MX2*	*OAS1*
Pregnant	10	0.71 ± 0.10 ^a^	0.28 ± 0.03 ^a^	0.64 ± 0.09 ^a^	0.66 ± 0.19
Non-pregnant	23	0.20 ± 0.05 ^b^	0.13 ± 0.02 ^b^	0.25 ± 0.06 ^b^	0.37 ± 0.06
Non-pregnant with late embryonic death	7	0.26 ± 0.12 ^b^	0.15 ± 0.03 ^b^	0.27 ± 0.09 ^b^	0.18 ± 0.04

Values are shown as copy number of gene/*GAPDH* (average ± SE). Different letters indicate significant differences between the pregnant, non-pregnant, and non-pregnant with late embryonic death groups based on Kruskal–Wallis test followed by Steel–Dwass test (*p* < 0.05). *ISG15*, interferon-stimulated gene 15; *MX1* and *MX2*, MX dynamin like GTPases 1 and 2; *OAS1*, 2’-5’ oligoadenylate synthetase 1; *GAPDH*, glyceraldehyde-3-phosphate dehydrogenase.

**Table 3:** Area under the curve (AUC) and the Youden index cutoff values estimated using receiver operating characteristic analysis in embryo-transferred JB cattle on day 21 of gestation (14 days after embryo transfer).

Value	*ISG15*	*MX1*	*MX2*	*OAS1*
AUC	0.903	0.883	0.883	0.623
Youden index	0.386	0.174	0.380	0.986

AUC values were considered high when 0.9 < AUC < 1, moderate when 0.7 < AUC < 0.9, low when 0.5 < AUC < 0.7, and nonpredictive when AUC < 0.5 [36,37].

**Table 4:** Predicted values on day 21 of gestation (14 days after embryo transfer) estimated using AVE and the Youden index in JB cattle.

	Threshold Value = AVE				Threshold Value = Youden Index			
	*ISG15*	*MX1*	*MX2*	*OAS1*	*ISG15*	*MX1*	*MX2*	*OAS1*
TP	10	10	10	6	8	9	9	4
FP	11	27	19	10	5	6	6	0
FN	0	0	0	4	2	1	1	6
TN	19	3	11	20	25	24	24	30
Sensitivity ^a^	100	100	100	60.0	80.0	90.0	90.0	40.0
Specificity ^b^	63.3	10.0	36.7	66.6	83.3	80.0	80.0	100
Accuracy	72.5	32.5	52.5	65.0	82.5	82.5	82.5	85.0
PPV	47.6	27.0	34.5	37.5	61.5	60.0	60.0	100
NPV	100	100	100	83.3	93.6	96.0	96.0	83.3

AVE, average value; TP, number of true positive cows; FP, number of false positive cows; FN, number of false negative cows; TN, number of true negative cows; PPV, positive predictive value; NPV, negative predictive value. The average values of the ISGs during the estrous cycle (Yoshino et al., 2020) and the Youden index were used as threshold values. Each value was estimated using the formula presented in the Materials and Methods Section. The sensitivity, specificity, accuracy, PPV, and NPV are shown as percentages. ^a^ Sensitivity indicates the probability that the cows that were truly pregnant were diagnosed as pregnant. ^b^ Specificity indicates the probability that the cows were diagnosed as non-pregnant.

## Data Availability

The datasets used and/or analyzed during the current study are available from the corresponding author on reasonable request.
